# MASLD: Spatial Mechanisms and New Therapeutics

**DOI:** 10.3390/biom16081129

**Published:** 2026-08-03

**Authors:** Christian Stoess, Janset Onyuru, Yanzhu Hu, Yuan Jiang, Zhengyi Xin, Aryan Panchal, Phillipp Hartmann

**Affiliations:** 1 Department of Surgery School of Medicine and Health, Technical University of Munich, TUM University Hospital, 81675 Munich, Germany; christian.stoess@tum.de (C.S.); janset.onyuru@tum.de (J.O.); yanzhu.hu@tum.de (Y.H.);; 2 Department of Pediatrics, University of California San Diego, 9500 Gilman Dr, La Jolla, CA 92093-0984, USA; 3 Division of Gastroenterology, Hepatology & Nutrition, Rady Children’s Hospital San Diego, San Diego, CA 92123, USA

**Keywords:** MASLD, MASH, liver zonation, hepatic fibrosis, hepatic stellate cells, pediatric MASLD, steatosis

## Abstract

Metabolic dysfunction-associated steatotic liver disease (MASLD) encompasses a spectrum of liver injury ranging from simple steatosis to metabolic dysfunction-associated steatohepatitis (MASH), a progressive inflammatory state associated with hepatocyte injury, fibrosis, cirrhosis, and hepatocellular carcinoma. Increasing evidence suggests that progression to MASH is not spatially uniform across the liver. Instead, it reflects region-specific metabolic stress, inflammatory signaling, and fibrogenic remodeling along the porto-central axis of the hepatic lobule. Recent advances in spatial transcriptomics, lipidomics, proteomics, and multiplex imaging have provided new insight into how hepatocytes, immune cells, endothelial cells, and hepatic stellate cells interact within distinct hepatic microenvironments during disease progression. In this review, we discuss emerging concepts linking hepatic zonation to steatosis, inflammation, fibrosis, and extracellular matrix remodeling in MASLD and MASH. We highlight how disruption of normal lobular organization contributes to a progressive loss of metabolic compartmentalization and to the amplification of inflammatory and fibrogenic signaling. We additionally examine how spatial disease programs differ between adult and pediatric MASLD, particularly given the periportal-predominant injury patterns frequently observed in children and the current lack of pediatric-focused mechanistic studies. Finally, we discuss recently approved therapies and emerging therapeutic strategies within the context of hepatic microenvironment remodeling and disease heterogeneity. Collectively, these findings support a framework in which MASLD and MASH are spatially organized diseases driven by dynamic multicellular interactions, emphasizing the importance of incorporating zonation and tissue context into future mechanistic studies and therapeutic development.

## 1. Introduction

### 1.1. Epidemiology

Metabolic dysfunction-associated steatotic liver disease (MASLD) has become the most prevalent chronic liver disease worldwide, currently affecting ~38% of adults globally and projected to exceed 55% by 2040, largely driven by rising rates of obesity and type 2 diabetes mellitus (T2DM) [1,2].

Recent global analyses underscore the rapid emergence of MASLD as a leading driver of liver-related morbidity and mortality, paralleling the worldwide expansion of obesity, insulin resistance, and metabolic syndrome [3]. Beyond its epidemiological scale, MASLD has become the leading indication for liver transplantation among women in the United States and a dominant underlying cause of hepatocellular carcinoma (HCC), surpassing hepatitis C in both contexts [2].

MASLD represents not only a hepatic disease but a systemic metabolic disorder, associated with cardiovascular disease, chronic kidney disease, sarcopenia, T2DM, and extrahepatic malignancies, highlighting the need for integrated, organ-system approaches in both research and clinical management [1,4].

The clinical spectrum of MASLD extends from simple hepatic steatosis to metabolic dysfunction-associated steatohepatitis (MASH), progressive fibrosis, cirrhosis, and ultimately to HCC [4]. However, disease progression is heterogeneous: only a subset of individuals with steatosis develop MASH, and the determinants of that transition, including genetic susceptibility, metabolic milieu, immune activation, and gut–liver axis dysfunction, remain incompletely understood [5].

A landmark international consensus process recently redefined the disease nomenclature, formally replacing the terms non-alcoholic fatty liver disease (NAFLD) and non-alcoholic steatohepatitis (NASH) with MASLD and MASH, respectively [6]. The updated framework, endorsed by the American Association for the Study of Liver Diseases (AASLD), the European Association for the Study of the Liver (EASL), and the Latin American Association for the Study of the Liver (ALEH), requires the presence of at least one cardiometabolic risk factor and introduces steatotic liver disease (SLD) as an umbrella term encompassing all causes of hepatic steatosis [6].

Despite major advances in understanding disease biology, effective treatments for MASH were limited and elusive until very recently, with the first U.S. FDA accelerated approvals emerging only in 2024 and 2025 for adult patients with at-risk MASH [7,8].

### 1.2. Pediatric MASLD

MASLD is increasingly recognized as one of the most common chronic liver diseases in children. Earlier epidemiologic studies estimated pediatric disease prevalence at approximately 5–10% in the general pediatric population in the United States, with substantially higher rates observed in children with obesity [9]. More recent global analyses suggest that pediatric MASLD prevalence may approach 10–13% among children and adolescents overall, while prevalence in children and adolescents with obesity may reach approximately 40–50%, with consistently higher prevalence reported in males [10,11].

These estimates are likely conservative, as pediatric MASLD remains substantially underdiagnosed due to limited awareness, inconsistent screening practices, and the lack of validated non-invasive diagnostic tools specifically calibrated for children [12].

Importantly, pediatric MASLD is not merely a precursor of adult disease but an early manifestation of a progressive metabolic disorder. Approximately 10–20% of children with MASLD evaluated in tertiary care settings may already exhibit advanced fibrosis at diagnosis [13]. Long-term follow-up from a Swedish national cohort demonstrated that individuals diagnosed with MASLD in childhood or young adulthood experience significantly higher risks of all-cause mortality, liver-related death, and cardiometabolic mortality compared with matched population controls, establishing that early-onset disease has lifelong consequences [14]. Consistent with these findings, MASLD has become one of the leading indications for liver transplantation among young adults in the United States [15].

The pathogenesis of pediatric MASLD reflects a convergence of developmental, genetic, and environmental factors that distinguish it mechanistically from adult-onset disease. Perinatal exposures, including maternal obesity, gestational diabetes, preterm birth, and early-life nutritional imbalance, can disrupt hepatic metabolic programming through mitochondrial dysfunction and epigenetic remodeling, increasing hepatic vulnerability [15]. Genetic risk variants, particularly the patatin-like phospholipase domain-containing protein 3 (*PNPLA3*) I148M allele and transmembrane 6 superfamily member 2 (*TM6SF2*) polymorphisms, further amplify susceptibility and disease severity, and these high-risk alleles are disproportionately prevalent in certain populations, including Hispanic children [14,15]. Environmental influences, including high dietary fructose intake, gut dysbiosis, and sedentary lifestyle, further exacerbate these vulnerabilities [16,17].

Despite the growing burden of pediatric MASLD and despite promising pharmacological interventions, no pharmacological therapy has yet been approved specifically for pediatric MASH, and the infrastructure required to conduct adequately powered pediatric histological trials remains limited [16,17].

### 1.3. Histological Scoring: The NASH CRN Criteria

Liver biopsy with standardized histological scoring remains the gold standard for diagnosing MASH and continues to serve as the primary endpoint in clinical trials. The NASH Clinical Research Network (NASH CRN) scoring system provides a validated semi-quantitative framework assessing the three cardinal histological features of MASH: hepatic steatosis (0–3), lobular inflammation (0–3), and hepatocellular ballooning (0–2) [18]. These components are combined to generate the NAFLD Activity Score (NAS), which ranges from 0 to 8. Scores ≥ 5 are generally consistent with active steatohepatitis, whereas scores ≤ 2 are unlikely to represent MASH [18]. Fibrosis is assessed on a scale from 0 to 4, with stages 3 and 4 (bridging fibrosis and cirrhosis) being the most strongly linked to long-term liver-related morbidity and mortality [19,20].

However, a key limitation of NAS-based scoring is that adult and pediatric MASH exhibit distinct histological phenotypes that reflect different spatial disease programs. Adult MASH (Type 1) is characterized by centrilobular (zone 3) steatosis, hepatocellular ballooning, Mallory-Denk body formation, and pericellular “chicken-wire” fibrosis, the features for which NAS criteria were originally developed [18]. In contrast, pediatric MASH (Type 2) typically demonstrates periportal (zone 1) steatosis, portal inflammation, and portal fibrosis with minimal hepatocellular ballooning [21]. Consequently, standard NAS criteria incompletely capture the pediatric disease phenotype, and histological endpoints derived from adult trials cannot be directly extrapolated to pediatric populations [12]. These differences are not merely descriptive but reflect fundamentally distinct hepatic microenvironments and zone-specific disease programs [15].

## 2. Metabolic Drivers of MASLD and Progression to MASH

Although obesity, insulin resistance, steatosis, genetic susceptibility, and gut dysbiosis are systemic drivers of MASLD, their hepatic effects are shaped by the porto-central architecture of the liver lobule [5,22]. The following sections therefore interpret these drivers as inputs into zone-specific hepatocyte stress, periportal inflammatory exposure, and fibrogenic remodeling, providing a bridge to the zonation-centered mechanisms discussed in Section 3.

### 2.1. Obesity

Obesity is one of the strongest and most modifiable drivers of MASLD initiation and progression. A large population-based study found that obesity in young adulthood, specifically around the age of 25, is strongly linked to the later development of MASLD, at-risk MASH, and increased liver stiffness between the ages of 40 and 80 [23]. Individuals who remained obese from young adulthood into later life had about a 5.47-fold increased risk of developing MASLD and were significantly more likely to display high-risk MASH and fibrosis-related phenotypes [23]. Duration of obesity exposure, rather than obesity itself, independently predicted progression of fibrosis and liver stiffness, whereas sustained weight loss significantly reduced these risks [23].

Among the metabolic features associated with MASLD, central adiposity and the expansion of visceral adipose tissue (VAT) are especially pathogenic. Compared with peripheral fat stores, VAT releases free fatty acids (FFAs) more readily and secretes pro-inflammatory adipokines that amplify hepatic lipid influx and systemic metabolic dysfunction [2]. These observations reinforce the concept that MASLD represents a systemic metabolic disease rather than a purely liver-restricted disorder, with strong bidirectional associations with cardiovascular disease, T2DM, and chronic kidney disease [2,24,25].

Obesity-driven expansion and dysfunction of adipose tissue further amplify hepatic injury through systemic inflammatory signaling. Dysfunctional VAT releases tumor necrosis factor alpha (TNF-α), interleukin-6 (IL-6), and pro-inflammatory chemokines that prime the liver’s innate immune system, activating Kupffer cells and sustaining a state of chronic low-grade inflammation [2,26]. These inflammatory signals, together with ongoing metabolic stress, activate hepatic stellate cells (HSCs) and engage transforming growth factor beta (TGF-β)-mediated fibrogenic cascades, initiating collagen deposition and the early structural remodeling that underlies fibrosis progression [26,27].

However, not all patients with MASH have obesity. A clinically important subset, commonly referred to as lean MASH, presents with a body mass index (BMI) within the normal range but often exhibits pronounced metabolic abnormalities, including dyslipidemia and increased VAT accumulation [2,28]. This phenotype underscores that the key determinants of disease progression are not adiposity per se but rather the overall pattern of metabolic dysfunction and fat distribution [28,29].

Lean MASH shares core pathogenic mechanisms with obesity-associated disease, including hepatic lipid accumulation, lipotoxicity, and insulin resistance, but several features distinguish it [30]. Many lean patients harbor central adiposity with increased VAT despite a normal BMI, resulting in elevated FFA flux to the liver [5,23,24,30,31]. Lean MASH is also frequently associated with sarcopenia, an independent predictor of fibrosis progression, and with an increased prevalence of extrahepatic malignancies, including lung and colorectal cancers [32,33].

In summary, obesity drives MASLD progression primarily through increased FFA delivery and adipose-derived systemic inflammation, while the lean MASH phenotype confirms that metabolic dysfunction, rather than body weight alone, is the operative driver of disease.

### 2.2. Insulin Resistance

Besides obesity, type 2 diabetes mellitus (T2DM) and insulin resistance are among the most important drivers of MASLD [2,34]. In children, insulin resistance frequently precedes overt T2DM and arises in the context of obesity, accelerating hepatic injury from an early age [35].

Insulin resistance, often accompanied by hyperinsulinemia and hyperglycemia in T2DM, disrupts systemic metabolic homeostasis by reducing glucose uptake in skeletal muscle, weakening insulin’s lipolytic effect in adipose tissue, and deregulating hepatic substrate metabolism. This coordinated dysfunction increases circulating glucose and FFAs, promotes chronic low-grade inflammation and ectopic lipid deposition, and drives steatosis, metabolic stress, and progressive liver injury [17,22,34,36,37]. Insulin resistance should be regarded as a primary pathogenic mechanism in MASLD rather than a simple metabolic correlate of obesity, because it converts excess adiposity into hepatocellular lipid excess, inflammatory signaling, and progressive tissue remodeling [34,35].

One of the earliest metabolic changes in this process occurs in adipose tissue, where loss of insulin-mediated suppression of lipolysis increases FFA flux to the liver and promotes hepatic triglyceride accumulation [38,39]. Hepatic insulin resistance then creates a selective metabolic imbalance: insulin no longer effectively suppresses gluconeogenesis, whereas hyperinsulinemia and hyperglycemia continue to activate sterol regulatory element-binding protein 1c (SREBP-1c)- and carbohydrate-responsive element-binding protein (ChREBP)-dependent lipogenesis, sustaining de novo lipogenesis (DNL) and hepatic lipid deposition [35,40]. Defective insulin signaling compromises fatty acid oxidation and mitochondrial adaptability, aggravating lipid retention and hepatocellular stress [35].

The link between insulin resistance and liver injury extends beyond hepatocytes [37,38]. Along the liver–adipose axis, complete hepatic insulin resistance does not invariably lead to steatosis under all dietary conditions, indicating that the traditional model of selective hepatic insulin resistance is incomplete [38]. Instead, hepatic endocrine signaling can propagate systemic metabolic dysfunction: in the insulin-resistant liver, forkhead box protein O1 (FoxO1) activation increases follistatin secretion, and circulating follistatin worsens adipose insulin resistance and lipolysis, further increasing FFA delivery to the liver [38].

Persistent insulin resistance also drives gut–liver axis dysfunction [41,42,43,44,45]. Excessive FFA influx, enhanced lipogenesis, and mitochondrial dysfunction activate NF-κB and the NOD-like receptor leucine-rich repeat and pyrin domain containing 3 (NLRP3) inflammasome, producing TNF-α, IL-6, and IL-1β, all of which can aggravate hepatocyte injury and impair insulin receptor signaling further [42,46]. The inflammatory effects also increase intestinal permeability, allowing lipopolysaccharide (LPS) and other microbial products to enter the portal circulation and activate hepatic TLR4 signaling in the liver. Thereby, a vicious cycle is being driven, promoting macrophage polarization, adipokine imbalance, and worsening glucose and lipid dysregulation [22,42,43,44]. Microbiota-derived metabolites contribute directly: propionate promotes insulin resistance via glucagon and fatty acid-binding protein 4 (FABP4), bile acid signaling through farnesoid X receptor (FXR) and Takeda G protein-coupled receptor 5 (TGR5) strongly influences glucose and lipid metabolism, and is linked to fibrosis progression [22,47,48], while indole derivatives improve fat metabolism and attenuate inflammation [49].

### 2.3. Steatosis

Hepatic steatosis is the defining pathological feature of MASLD and represents the earliest stage of disease development [50]. Steatosis is commonly defined histologically as fat accumulation in >5% of hepatocytes, or by imaging-based estimates of hepatic fat content [51]. It reflects an imbalance between lipid acquisition and disposal within hepatocytes [52], arising from increased fatty acid uptake, enhanced DNL, impaired fatty acid oxidation, and reduced lipid export as very-low-density lipoproteins (VLDL). DNL is particularly relevant, as excess carbohydrates are converted into fatty acids and esterified into triglycerides, with acetyl-CoA carboxylase (ACC) and fatty acid synthase (FAS) playing central roles [52,53]. Excessive fructose consumption is particularly pathogenic because, unlike glucose, fructose bypasses regulatory checkpoints after hepatic uptake and enters the DNL pathway in an unregulated manner, driving fatty acid synthesis and hepatic lipid accumulation [53,54]. Fructose metabolism also generates large quantities of metabolic intermediates that enter the tricarboxylic acid (TCA) cycle, increasing citrate export and providing additional acetyl-CoA for lipid synthesis [54]. The adenosine triphosphate (ATP) consumption required for fructose phosphorylation induces cellular energy depletion and triggers oxidative and metabolic stress that promotes inflammatory signaling, accelerating the progression from steatosis to steatohepatitis [5,53].

Not all fatty acids exert equal lipotoxic effects. Saturated fatty acids (SFAs), particularly palmitate and stearate, are substantially more hepatotoxic than monounsaturated fatty acids (MUFAs) or polyunsaturated fatty acids (PUFAs). SFAs induce endoplasmic reticulum (ER) stress, activate the Toll-like receptor 4-c-Jun N-terminal kinase (TLR4-JNK) inflammatory axis, promote ceramide synthesis, and can directly activate HSCs to initiate pro-fibrotic signaling [5,25]. MUFAs attenuate lipotoxicity by facilitating storage of excess lipid in a less toxic form and by modulating membrane lipid composition [25,27].

At the organelle level, early MASLD is characterized by a compensatory increase in mitochondrial β-oxidation and TCA cycle activity in response to excess substrate delivery. As the disease progresses, however, mitochondria develop structural and functional abnormalities, including swelling, disrupted cristae architecture, and reduced oxidative capacity, which impair hepatocyte metabolic flexibility and promote excessive reactive oxygen species (ROS) production, exacerbating oxidative stress [28,55]. Concurrent endoplasmic reticulum (ER) stress and impaired protein folding activate nuclear factor-kappa B (NF-κB) and related inflammatory pathways, further amplifying hepatocellular injury [55,56]. As metabolic stress persists, these pathways increasingly engage inflammasome-associated programs of inflammatory cell death, further amplifying inflammatory signaling during MASH progression [57]. Hepatocyte injury, immune cell recruitment, and activation of fibrogenic pathways ultimately drive progression from MASLD to inflammatory MASH [5,28,58].

Together, these stress pathways progressively impair hepatocellular homeostasis and reduce the liver’s capacity to adapt to ongoing metabolic overload.

Once hepatocellular injury occurs, steatosis drives disease progression by altering the hepatic microenvironment. Damage-associated molecular patterns (DAMPs) released from damaged or dying hepatocytes activate hepatic immune cells and amplify inflammatory responses, promoting recruitment and activation of macrophages and other immune cell populations. In parallel, stress signals and inflammatory mediators activate HSCs, which produce extracellular matrix (ECM) components and initiate fibrogenesis [59].

The biological consequences of steatosis are further shaped by factors beyond the hepatocyte itself: through the gut–liver axis, microbiota-derived metabolites modulate hepatic lipid metabolism, fatty acid oxidation, and inflammation, influencing both the degree and consequences of steatosis. Host genetic background additionally determines susceptibility and progression [60]. Together, persistent metabolic stress and inflammatory signaling shift the hepatic environment from benign lipid accumulation toward hepatocyte injury, immune cell recruitment, and fibrogenic activation, ultimately driving progression from MASLD to inflammatory MASH [5,58].

### 2.4. Genetic Susceptibility

Genetic background shapes individual susceptibility to obesity-driven MASLD across each of the mechanisms described above. Variants in *PNPLA3*, *TM6SF2*, membrane-bound O-acyltransferase domain-containing 7 (*MBOAT7*), and glucokinase regulator (*GCKR*) influence hepatic lipid metabolism, lipid droplet remodeling, and lipoprotein secretion, increasing the risk of MASH and liver fibrosis in genetically predisposed individuals [5,29]. Genetic susceptibility plays a particularly prominent role, most notably the *PNPLA3* I148M variant, which strongly promotes hepatic fat accumulation and fibrosis risk even in the absence of adiposity [30].

These inherited variants do not act in isolation. Host genotype and gut microbial composition influence one another, and genetic risk for MASLD is increasingly understood to be modified by the intestinal microbiome, a relationship explored in detail below.

### 2.5. Gut Dysbiosis

Following obesity-associated insulin resistance and hepatic steatosis, gut dysbiosis emerges as a major driver of hepatic inflammation and fibrosis. In MASLD, dysbiosis is not limited to a simple compositional shift in intestinal microbes but instead comprises a coordinated disturbance involving increased microbial burden, reduced microbial diversity, domination by pathogenic taxa, altered metabolite output, and disruption of intestinal barrier integrity. Multi-omics analyses combining gut microbiota composition and metabolite profiles, including short-chain fatty acids (SCFAs), provide complementary, non-invasive signals of MASLD progression and severity [61,62]. At the community level, anti-inflammatory and short-chain fatty acid (SCFA)-producing taxa, including members of *Ruminococcaceae*, *Alistipes*, *Blautia*, and *Faecalibacterium*, are reduced, whereas disease-associated organisms such as *Escherichia* and other potentially proinflammatory microbes become enriched [22,43,61,63]. This is associated with increased translocation of bacterial LPS, endogenous ethanol, and trimethylamine N-oxide (TMAO) into the bloodstream, and dysregulation of bile acid, amino acid, and SCFA metabolism, all of which exacerbate MASLD [62,64]. This ecological imbalance also extends beyond bacteria, because reduced fungal diversity and increased *Candida* abundance have been demonstrated in MASH or advanced fibrosis [65]. Although fungal aberrations, including an increased relative abundance of the genus *Candida* and the species *Candida albicans*, have been identified in various liver diseases [66], specific fungal signatures have been characterized to differentiate MASLD from other liver diseases, including alcohol-associated liver disease [65,67]. Further, the viral microbiome has been investigated, and reduced gut viral diversity together with depletion of selected *Lactococcus* phages and *Leuconostoc* phages, as well as increased abundance of selected *Lactobacillus* phages, have been described in advanced MASLD [68,69]. Accordingly, gut dysbiosis is best interpreted as a disorder that links simple steatosis to the persistent inflammatory activity characteristic of MASH.

A principal consequence of dysbiosis is impairment of intestinal barrier homeostasis and the resulting increase in portal delivery of microbial products to the liver.

Loss of beneficial SCFA-producing bacteria reduces butyrate generation and weakens epithelial support, while Gram-negative overgrowth increases LPS exposure and promotes degradation of tight-junction components, including occludin, claudin-1, zonula occludens 1 (ZO-1), and junctional adhesion molecule A (JAM-A), thereby increasing intestinal permeability and favoring a leaky gut phenotype [43,70,71]. In parallel, reduced abundance of taxa involved in mucosal maintenance further diminishes host defense at the intestinal interface, and this effect is particularly relevant in fibrotic disease, where depletion of butyrate-associated organisms is accompanied by signatures of impaired barrier protection [72]. Once the barrier is compromised, LPS and other pathogen-associated molecular patterns (PAMPs) enter the portal circulation more readily and reach the liver, where they activate Kupffer cells and other immune cells [64]. Supernatant from feces of patients with fibrosis decreases epithelial monolayer resistance, and this effect is partially reversible with protease inhibition, supporting a direct contribution of microbiota-derived protease activity to barrier disruption [72]. These observations indicate that increased intestinal permeability is a central intermediate step through which dysbiosis translates luminal microbial imbalance into a hepatic inflammatory burden.

After gut-derived inflammatory signals enter the liver, the progression toward steatohepatitis and fibrosis is shaped by a combination of innate immune activation and failure of intrahepatic immunoregulatory mechanisms. A central mechanism in this process involves LPS-driven activation of TLR4 and TLR9, which, in turn, promotes NF-κB signaling and inflammasome engagement, thereby sustaining the production of proinflammatory mediators that aggravate hepatocellular injury and fibrogenesis [70,71,73]. These abnormalities become increasingly pronounced during progression from simple steatosis to steatohepatitis and fibrosis [73,74]. In alignment with this model, Henao-Mejia et al. found that deficiencies in NLRP3 or NLRP6, as well as loss of the downstream cytokine IL-18, lead to the development of a transmissible dysbiotic microbiota. This results in elevated portal delivery of microbial products and increased hepatic TNF-α expression, thereby accelerating the advancement of MASLD and MASH [73]. In addition, inflammatory signaling prompted by dysbiosis plays a critical role in aggravating insulin resistance, as LPS and related mediators interfere with insulin signaling pathways and intensify the relationship between metabolic dysfunction and inflammation [73,74,75]. Within this pathological context, HSCs are not merely collagen-producing fibrogenic effector cells, but also important regulators of the hepatic immune microenvironment. By releasing mediators such as bone morphogenetic protein 9 (BMP9), BMP10, colony-stimulating factor 1 (CSF1), and IL-34, HSCs support the maintenance and identity of Kupffer cells, and suppressor of cytokine signaling 1 (SOCS1)-dependent pathways within HSCs further limit excessive inflammatory activation. Once these homeostatic functions are compromised, resident Kupffer cells are progressively replaced by pro-inflammatory monocyte-derived macrophages, which favor persistent tissue damage and accelerate fibrogenic progression [76]. Hence, continuous exposure to gut-derived inflammatory signals disrupts the intrahepatic macrophage niche and sustains stellate cell activation, leading to persistent hepatic immune dysregulation in MASH [74].

Beyond elevated serum LPS levels, or endotoxemia, dysbiosis alters the intestinal metabolite pool, further aggravating lipid accumulation, oxidative stress, and immune activation. Reduced SCFA production is a recurring feature across studies. It has dual pathological relevance, because SCFAs support epithelial integrity while also regulating hepatic energy metabolism, partly through AMPK-related pathways and mitochondrial fatty acid oxidation [62]. Dysbiosis is also associated with bile acid remodeling. Enrichment of organisms such as *Desulfovibrio* increases secondary bile acids, including deoxycholic acid (DCA) and lithocholic acid (LCA), suppresses FXR signaling, impairs fatty acid β-oxidation, and promotes hepatic lipid accumulation [49,71]. In parallel, dysregulated bile acid metabolism reduces TGR5 activation and glucagon-like peptide-1 (GLP-1) secretion while relieving inhibition of cytochrome P450 family 7 subfamily A member 1 (CYP7A1), together promoting bile acid overproduction, insulin resistance, and hepatic steatosis [70]. Consistent with these findings, fibrosis-associated signatures such as increased glycoursodeoxycholic acid 3-sulfate (G-UDCA-3S) and an inverse relationship between *Tuzzerella* and glycine-conjugated bile acids suggest that bile acid disturbances become more pronounced with disease progression [72]. In parallel, microbial metabolites such as TMAO are implicated in MASLD pathogenesis and are linked to mitochondrial dysfunction, inflammation, endothelial injury, and fibrosis-related pathways [43,49,71].

Loss of protective tryptophan-derived microbial metabolites provides an additional mechanism linking dysbiosis to progression from MASLD to MASH. Levels of indole derivatives such as indole-3-propionic acid (IPA) and indole-3-acetic acid (IAA) are reduced in MASLD, despite their normal contribution to gut–liver homeostasis [49]. Both metabolites suppress TLR4/NF-κB signaling, reduce endotoxin translocation, and limit hepatic macrophage activation [49]. As an IAA-producing bacterium, *Bifidobacterium bifidum* enhances aryl hydrocarbon receptor (AHR)-dependent intestinal signaling and reduces circulating LPS-binding protein (LBP) together with hepatic C-C motif chemokine ligand 2 (CCL2) and CCL5 expression, consistent with attenuation of macrophage infiltration and inflammatory progression [49].

Intestinal microbes can metabolize unabsorbed fructose into acetate, and selected bacteria generate ethanol through carbohydrate fermentation [46]. Microbial ethanol reaches the liver through the portal circulation. It is converted by alcohol dehydrogenase (ADH) and cytochrome P450 2E1 (CYP2E1) into acetaldehyde, promoting oxidative stress, lipid peroxidation, and inflammation. In contrast, acetate can be converted by acyl-CoA synthetase short-chain family member 2 (ACSS2) into acetyl-CoA and contribute to DNL and hepatic triglyceride accumulation [46,70]. Portal ethanol concentrations are markedly elevated in MASH compared with obesity-associated steatosis, and this difference is abolished by antibiotic treatment, further implicating microbial ethanol production in disease progression [77]. Intestinal fructose metabolism may still provide sufficient substrate for excessive triacylglycerol formation even when liver-specific fructokinase pathways are impaired, indicating that under conditions of hepatic insulin resistance, intestinal metabolism can bypass part of the hepatic enzymatic constraint and continue to sustain lipid accumulation [46].

## 3. Liver Zonation in MASLD-MASH and Cellular Interactions

### 3.1. MASLD and MASH Zonation

To study liver zonation, researchers have historically relied on histological techniques and immunostaining of selected proteins, potentially biasing investigations toward already known pathways. Advances in spatially resolved technologies, including mass spectrometry (MS)-based proteomics, spatial transcriptomics, and multiplex immunofluorescence, now enable unbiased characterization of hepatic zonation and cellular interactions [78].

Recent discoveries have enhanced our understanding of the liver’s three-dimensional organization and how anatomical structure and physiological conditions shape cellular function. Historically, the anatomical concepts of liver lobes and lobules have been well established and were derived from both macroscopic and microscopic observations [79]. On the histological level, liver lobules are organized as hexagonal units with periportal fields located at each corner. These periportal regions consist of branches of the hepatic artery, portal vein, and bile duct. Septa covering the liver surface extend deeper into the tissue and surround these three vessel types. Within this structure, blood flow and bile flow occur in opposite directions. Blood flows from the portal triads through the sinusoidal network toward the vein, whereas bile flows in the opposite direction toward the bile ducts. Thus, liver anatomy and physiology are closely interconnected and shape the functional specialization of liver cells, leading to the concept of liver zonation. Three distinct zones have traditionally been proposed. Zone 1, located closest to the periportal fields, receives blood with the highest oxygen content, and the sinusoidal fenestrae are largest in this region [80]. Functionally, hepatocytes in zone 1 express high levels of enzymes involved in gluconeogenesis, β-oxidation, and vitamin A metabolism. Zone 3, located adjacent to the central vein, contains hepatocytes that receive blood with lower oxygen concentrations and therefore specialize in glycolysis, lipogenesis, and triglyceride synthesis [80]. Although these observations have been established for decades, relatively little is known about how liver zonation influences cell types beyond hepatocytes or how these cells respond under pathological conditions. Given that zonation strongly affects liver metabolism, it is particularly important to understand how hepatocytes and other non-parenchymal cells, such as HSCs, behave during MASLD and MASH. Interestingly, the maintenance of this spatial organization is controlled by several conserved developmental pathways.

### 3.2. Developmental Signaling Pathways Shaping Zonation

**Figure 1:**
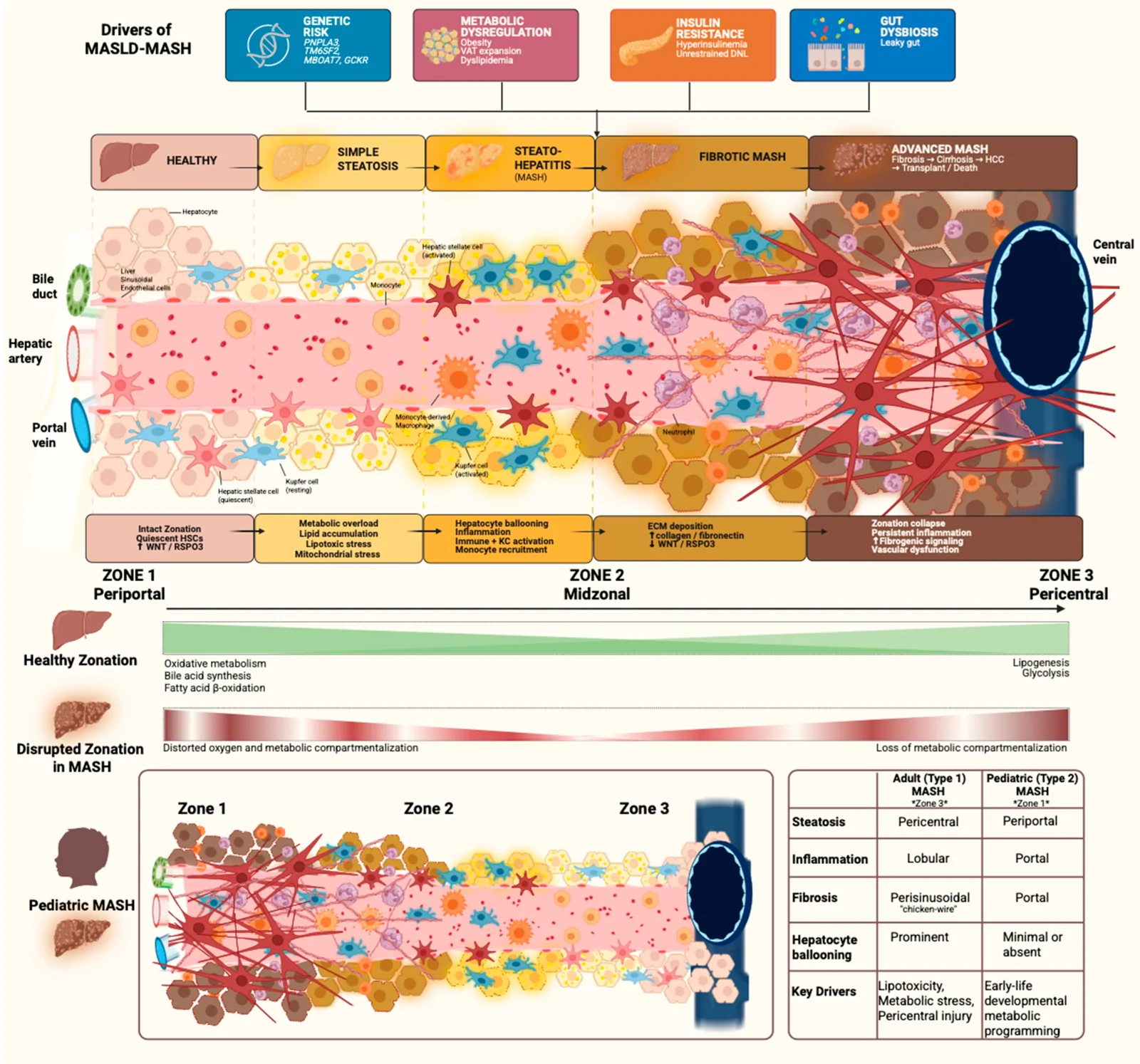
Spatial and cellular progression of MASLD-MASH across the hepatic lobule. Schematic overview of disease progression from healthy liver to advanced MASH across the porto-central axis. Genetic susceptibility, metabolic dysregulation, insulin resistance, and gut dysbiosis promote steatosis, inflammation, fibrosis, and progressive disruption of hepatic zonation. Disease progression is associated with hepatocyte injury, immune-cell recruitment, HSC activation, ECM deposition, and zonation collapse, ultimately contributing to cirrhosis and HCC. The lower panel highlights distinct spatial disease programs in adult and pediatric MASH. Adult MASH (Type 1) is characterized by predominant zone 3 (pericentral) steatosis, lobular inflammation, hepatocyte ballooning, and perisinusoidal fibrosis. Pediatric MASH (Type 2) exhibits a zone 1 (periportal) pattern characterized by periportal steatosis, portal inflammation, portal fibrosis, and minimal hepatocyte ballooning. Horizontal arrows (→) indicate disease progression. Upward arrows (↑) indicate an increase, whereas downward arrows (↓) indicate a decrease. Asterisks (*) indicate predominance rather than certainty of the zonation trend. Created with a license from biorender.com.

Recent studies have shown that developmental pathways responsible for establishing normal liver zonation are reactivated in metabolic liver disease (Figure 1). These include Wnt/β–catenin, Notch, Hedgehog, and Yap/Taz/Hippo signaling pathways, which have been implicated in obesity-associated MASH and fibrosis progression [81,82]. In this context, the Wnt/β–catenin pathway plays a key role in hepatic zonation by orchestrating distinct functions of liver metabolism and modulating the expression of ca. 30% of zonated genes. β-catenin is the central actor in the Wnt/β–catenin signaling cascade, and its activation is crucial for the metabolic patterning of the liver [82]. In addition, key metabolic pathways involved in MASH pathogenesis, including peroxisome proliferator-activated receptor (PPAR) and FXR signaling, also display zonal patterns, highlighting the importance of metabolic organization in disease development. Hepatocyte nuclear factor 4 alpha (HNF4α) suppresses the expression of pericentral proteins in periportal hepatocytes, possibly through a histone deacetylase 1 (HDAC1)-mediated mechanism [22]. Epigenetic mechanisms may further contribute to zonation. Differential methylation of transcription factor binding sites can generate spatially restricted activity of transcription factors that are uniformly expressed, including HNF4A and retinoid X receptor (RXR). In the periportal zone, enrichment of genes associated with Notch signaling pathways has been observed, whereas Wnt signaling is enriched among genes upregulated pericentrally [83]. Interestingly, classical Wnt agonists such as WNT3A and WNT3, as well as components of the export machinery, including porcupine (PORCN) and Wntless (WLS), are not strongly zonally expressed. This observation suggests that the pericentral Wnt gradient may arise not primarily from ligand distribution but from differential sensitivity of hepatocytes to Wnt signals. Negative regulators of Wnt signaling, such as palmitoleoyl-protein carboxylesterase NOTUM homolog (NOTUM), naked cuticle homolog 1 (NKD1), ring finger protein 43 (RNF43), and zinc and ring finger protein 3 (ZNRF3), are upregulated pericentrally, creating a tightly controlled signaling environment. Consistent with this idea, Wnt signaling enhancers such as *R-spondin (RSPO) 2*, *RSPO3*, and *Leucine-rich repeat-containing G protein-coupled receptor 5 (LGR5)* are expressed at substantially higher levels pericentrally than periportally, suggesting increased sensitivity of pericentral hepatocytes to canonical Wnt signals and underscoring the pronounced zonation patterns exhibited by hepatocytes within the liver [83,84]. Emerging evidence suggests that Hedgehog (Hh) signaling contributes to hepatic zonation in addition to its roles in liver development and regeneration [85]. In the healthy adult liver, Hh signaling is relatively quiescent in hepatocytes but remains more active in HSCs and cholangiocytes. In contrast to canonical Wnt/β–catenin signaling, which predominates in the pericentral region, Hh signaling is enriched in the periportal compartment and has been implicated in regulating zone 1 hepatocyte identity and function [86]. Notably, Hh pathway activity is reactivated during chronic liver injury, including MASLD, cirrhosis, and hepatocellular carcinoma, promoting HSC activation, ductular reactions, and fibrogenic remodeling [87]. These findings suggest that dysregulated Hh signaling may contribute to the disruption of normal hepatic zonation during disease progression and highlight this pathway as a potential source of diagnostic biomarkers and therapeutic targets.

### 3.3. Hepatocyte Zonation and Metabolic Specialization

Single-nucleus RNA sequencing of a porcine MASLD model identified zonated transcriptional states in hepatocytes and endothelial cells, with hepatocytes classified into periportal, mid-lobular, and pericentral populations [88]. Periportal hepatocytes showed higher expression of genes associated with protein and lipoprotein synthesis, including *albumin* (*ALB*), *apolipoprotein* (*APO) A*1, and *APOC2*; mid-lobular hepatocytes expressed *phosphoenolpyruvate carboxykinase 1* (*PCK1*), *glucose-6-phosphatase catalytic subunit 1* (*G6PC*), *hepatic lipase* (LIPC) and *Arginase 1* (*ARG1*), consistent with glucose metabolism, lipolysis, and ureagenesis; and pericentral hepatocytes were characterized by *metallothionein 1A* (*MT1A*) and detoxification-associated genes [78,89,90].

However, single-cell spatial reconstruction of mouse liver showed that hepatocyte gene expression is organized along a continuous porto-central axis, with many genes displaying gradual gradients or non-monotonic patterns that peak in mid-lobular regions [89,91].

Spatial transcriptomic analysis of mouse liver has also identified periportal, mid-lobular, and pericentral hepatocyte populations using established zonal markers, including *Pck1*, *histidine ammonia-lyase* (*Hal)*, *glutaminase 2* (*Gls2)*, *hydroxysteroid 17-beta dehydrogenase 13* (*Hsd17b13)*, and *argininosuccinate synthase 1* (*Ass1)* in periportal regions [92]. 

In healthy human liver, spatial transcriptomics identified periportal landmark genes including *ALB*, *APOA1*, *ALDOB*, *ASS1*, *HAL*, and *serine dehydratase* (*SDS*), whereas pericentral landmark genes included *CYP2E1*, *CYP1A2*, *CYP3A4*, and *ornithine aminotransferase (OAT)* [91,93].

Combined transcriptomic and DNA methylation mapping of microdissected human liver further identified anti-correlated expression and methylation gradients across periportal, intermediate, and pericentral regions, including a prominent pericentral Wnt-associated epigenetic signature [83]. 

Liver transcriptional heterogeneity is predominantly driven by lobular zonation, a pattern confirmed across independent spatial transcriptomic datasets [94]. At the protein level, single-cell spatial proteomics showed that zonated metabolic pathways are more strongly conserved between human and mouse liver than individual zonated proteins [95]. Ribosomal proteins were consistently enriched periportally in human liver, whereas this pattern was not observed in mice. Pericentral hepatocytes appear particularly dependent on oxygen and Wnt signaling for maintaining their zonated identity and function, potentially explaining their increased vulnerability to injury [78,95]. 

Zonation also extends to non-parenchymal cell (NPC) populations, as discussed in the following section.

### 3.4. Zonation of Non-Parenchymal Cells (NPCs)

To date, a comprehensive comparison between physiological liver zonation and zonation in MASLD remains incomplete, particularly for NPC. This is largely due to the predominance of hepatocytes within the liver, which complicates the spatial characterization of less abundant NPC populations and obscures their heterogeneity. Besides hepatocytes, endothelial cells also exhibit zonation. Central vein endothelial cells express WNT2, WNT9b, CDH13, and RSPO3, whereas periportal endothelial cells express von Willebrand factor (vWF) [96]. Liver sinusoidal endothelial cells (LSECs) show both periportal and pericentral transcriptional signatures. Periportal LSECs displayed increased intercellular adhesion molecule-1 (ICAM1) expression [95]. In contrast, stellate cells and macrophages may not follow the same smooth, porto-central zonation observed in hepatocytes, though disease-associated stellate cell and macrophage niches can still be highly spatially organized. For instance, HSCs can be profiled as central vein- or portal vein-associated. In a mouse carbon tetrachloride (CCl_4_) model, the dominant pathogenic collagen-producing HSCs are located around the central vein [97]. In an inflamed liver, activated HSCs can be characterized by increased actin alpha 2 (ACTA2) expression and activation of mitogen-activated protein kinase (MAPK), Ras-proximate-1 (Rap1), and focal adhesion pathways, alongside reduced retinol metabolism. Upon metabolic liver injury, macrophages shift toward a cluster of differentiation 68 (CD68)-high, major histocompatibility complex class II (MHC-II)-high inflammatory phenotype with increased lysosomal and phagocytic gene expression [88]. Periportal KCs produce more TNF-α and PGE, while KCs located closer to the central vein produce more Il-1, suggesting a functional zonation of macrophage subsets. However, these zonation properties might be affected by the decrease in healthy Kupffer cells in MASLD, where bone marrow-derived macrophages replace them [98]. These recruited macrophages exist in two distinct subsets, either resembling homeostatic Kupffer cells or lipid-associated macrophages (LAMs), as observed in obese adipose tissue [99]. Beyond intrinsic hepatocyte programs, stromal cells also contribute to maintaining zonal metabolic identity.

### 3.5. Stromal Regulation of Metabolic Zonation

Recent studies have demonstrated that the surrounding stromal niche can regulate hepatocyte-intrinsic metabolic programs. HSCs have emerged as key regulators of metabolic zonation by secreting the WNT signaling amplifier RSPO3. Loss of stellate cells or conditional deletion of RSPO3 disrupts hepatocyte Wnt/β–catenin signaling and alters zonal hepatocyte programs, resulting in contraction of pericentral metabolic signatures and expansion of periportal gene expression. These findings highlight the importance of stellate cells in regulating hepatocyte metabolism and maintaining liver homeostasis in multiple liver disease conditions, including MASH [100].

### 3.6. Lipid Metabolism and Zonation in MASLD/MASH

Recent spatial lipidomic studies demonstrate that MASH is associated with profound disruption of normal hepatic zonation. In a combined diet- and CCl_4_-induced mouse model, Seubnooch et al. identified 130 lipids with altered abundance and distribution across the liver lobule and showed that multiple lipid classes, including triacylglycerols, diacylglycerols, sphingolipids, and ceramides, undergo marked zone-specific remodeling during MASH. Notably, many lipid species shifted from predominantly pericentral localization in control livers toward periportal enrichment in MASH, indicating perturbation of normal zonal lipid organization. MASH-associated lipid accumulation was enriched in SFAs and MUFAs, whereas reduced lipid species were enriched in polyunsaturated fatty acids. The authors also observed increased periportal fibrosis, suggesting that altered spatial lipid metabolism may contribute to regional fibrogenic responses during disease progression. Together, these findings highlight that MASH is not only a disorder of bulk lipid accumulation but also one of profound spatial metabolic reprogramming across the liver lobule [101].

### 3.7. Zonation and Fibrosis Amplification

Glycolysis in HSCs contributes to liver fibrosis progression by promoting the release of fibrogenic extracellular vesicles (EVs). Genetic inhibition of glycolysis specifically in HSCs reduces fibrosis in vivo. Spatial transcriptomics has demonstrated that fibrosis increases EV-related pathways predominantly in the hepatic pericentral zone, and this effect is suppressed when glycolysis is inhibited. Mechanistically, glycolysis enhances histone H3 lysine 9 (H3K9) acetylation at the promoters of EV-associated genes such as RAB31, increasing EV production. These EVs stimulate fibrotic gene expression in recipient HSCs, thereby amplifying fibrosis. EVs derived from glycolysis-deficient mice lack this profibrotic activity. Overall, this study identified HSC glycolysis-dependent EV release in the pericentral zone as a mechanism promoting fibrosis amplification [102].

### 3.8. Remodeling of Zonation During Disease

Zonation patterns may change substantially during disease progression. Watson et al. identified three hepatocyte clusters in healthy human livers using multiplexed error-robust fluorescent in situ hybridization [103]. In fibrotic livers, however, only two zonated populations were observed: periportal hepatocytes and pericentral hepatocytes. In addition, two injury-associated hepatocyte clusters were identified that lacked zonation and were distributed throughout the lobule. These findings suggest that the classical three-zone model approximates a continuous gradient of gene expression and function. Zonal receptor and ligand expression patterns may also shape interactions between hepatocytes and NPCs. For example, thrombospondin signaling (THBS1/2) from HSCs to CD36-expressing zone-3 hepatocytes may influence MASLD pathogenesis, while WNT2B expressed by endothelial cells and macrophages interacts with frizzled class receptor 6 (FZD6) and LRP5/6 receptors on pericentral hepatocytes [103].

### 3.9. Limitations of Spatial Methodologies in MASLD/MASH Research

The advances described above should be interpreted alongside several methodological constraints affecting MASLD/MASH research more broadly. Liver biopsy remains the principal source of human tissue for conventional histology and spatial analyses but is inherently affected by sampling bias, as small biopsy cores may not capture the heterogeneous distribution of steatosis, inflammation, and fibrosis across the hepatic lobule. Non-invasive tools such as alanine aminotransferase (ALT), body mass index (BMI), magnetic resonance imaging-based proton density fat fraction (MRI-PDFF), and elastography reduce reliance on biopsy but were developed and validated mainly in adult cohorts, and their diagnostic thresholds may not generalize across the lifespan. This is particularly relevant in pediatric MASLD, where periportal-predominant injury pattern differs from the pericentral pattern typical of adult disease, tissue availability is limited, well-characterized biopsy cohorts remain scarce, and adult-derived biomarker and imaging thresholds have not been systematically re-validated in children (see Table 1). Preclinical models add further uncertainty, as animal systems do not fully recapitulate the zonal, immune, and metabolic architecture of human liver disease.

Technical factors specific to spatial platforms compound these constraints. Tissue fixation, processing, and preservation methods can affect RNA, protein, and lipid integrity, and available spatial technologies differ considerably in resolution, molecular coverage, and analytical sensitivity, complicating direct comparison across studies. Spatial lipidomic platforms carry an additional identification caveat because they often localize lipids based on mass rather than directly confirming molecular structure, making chemically distinct species with similar masses difficult to distinguish. Zone-specific lipid patterns should be interpreted as candidate spatial metabolic signatures unless validated using orthogonal MS/MS-based or targeted lipidomic approaches. Transcript abundance also does not necessarily predict protein expression or functional activity, underscoring the need for complementary proteomic and functional validation. 

Finally, translating spatial findings into clinically applicable biomarkers, therapeutic targets, and patient outcomes remains difficult, as associations with histological progression, treatment response, and long-term clinical endpoints, are still emerging, particularly in children.

## 4. New and Approved Therapeutics and Outlook

### 4.1. From Zonation-Aware Pathobiology to Therapeutic Logic

Progression from MASLD to MASH reflects a shift from isolated steatosis to spatially organized cellular stress, inflammation, and fibrogenesis.

Accordingly, therapies are grouped according to their primary biological targets in disease progression: (1) metabolic dysregulation, (2) inflammation, and (3) fibrogenesis and ECM remodeling. This mechanism-based grouping is retained but interpreted through spatial disease biology: metabolic therapies mainly address hepatocyte lipid stress and systemic metabolic overload, anti-inflammatory approaches target gut–liver immune traffic and innate immune activation, and antifibrotic strategies focus on stromal, vascular, and ECM remodeling. This framework links treatment mechanisms to hepatic microenvironments without implying that current drugs are zone-selective [76,111].

### 4.2. Trial Endpoints and Evidence Hierarchy: What Disease Modification Means in MASH

Due to the prolonged follow-up required to capture clinically meaningful liver outcomes, MASH trials continue to rely on biopsy-based endpoints for accelerated approval. The two standard endpoints remain (i) MASH resolution without worsening of fibrosis and (ii) fibrosis improvement by at least one stage without worsening of MASH. Fibrosis stage remains the histologic variable most strongly associated with long-term liver-related outcomes, which is why current regulatory and society frameworks continue to anchor pivotal development on histology-based readouts [19,24,104,112].

However, this endpoint framework should not be considered static. Early-phase and translational studies increasingly integrate dynamic non-invasive measures, including MRI-proton density fat fraction (PDFF), transient elastography (FibroScan) or magnetic resonance elastography, serum fibrosis panels, and composite response tools such as the MASH Resolution Index, to support dose selection, response enrichment, and mechanistic interpretation [105,111]. Disease modification in MASH is therefore best viewed as an evidence hierarchy in which regulatory histology remains the gold standard. In contrast, non-invasive biomarker trajectories and target-engagement readouts help determine whether a therapy plausibly modulates the intended metabolic or fibrogenic pathways [105,111,112].

In this review, we classify major therapeutic classes by their highest current evidence tier: Approved (regulatory approval for MASH), Phase III (pivotal histology trial completed), Phase II (proof-of-concept histology trial), and Early/Mechanistic (preclinical or first-in-human).

### 4.3. Approved Therapies: The Emergence of a Metabolic Backbone

The approvals of resmetirom and semaglutide have shifted MASH therapeutics from a pre-approval landscape to one defined by two complementary backbone strategies): a liver-directed approach targeting intrahepatic lipid metabolism and a systemic approach focused on whole-body metabolic improvement (Table 2). However, this distinction should not imply that either drug functions as a stand-alone antifibrotic therapy. Rather, both should be regarded as backbone therapies that address different, but complementary, aspects of this disease [7,111,112,113]. Additional agents Phase II development and other emerging therapeutic strategies are summarized in Table 3.

#### 4.3.1. Resmetirom (THR-β Agonist): Hepatoselective Lipid-Flux Reprogramming

Resmetirom is a liver-directed thyroid hormone receptor-β (THR-β) agonist that reduces hepatic lipid burden by enhancing fatty-acid oxidation. In MAESTRO-NASH, resmetirom 80 mg and 100 mg resulted in MASH resolution without worsening of fibrosis in 25.9% and 29.9% of patients, respectively, compared with 9.7% with placebo, and fibrosis improvement by at least one stage without worsening of disease activity in 24.2% and 25.9% versus 14.2% with placebo at week 52, all significantly statistically better than placebo [7]. Low-density lipoprotein cholesterol was reduced by 13.6% and 16.3% at week 24, reinforcing the view that resmetirom functions as a hepatoselective metabolic agent with clinically relevant lipid benefits [7,111]. Based on these findings, resmetirom was approved for patients with moderate-to-advanced fibrosis without cirrhosis due to MASH by both the FDA [114] and the European Union [115].

Mechanistically, THR-β agonism is well matched to the pericentral metabolic vulnerability characteristic of adult MASH, in which lipotoxic injury, hepatocyte ballooning, and perisinusoidal fibrosis are prominent [18]. From a combination perspective, THR-β agonists are plausible partners for (i) incretin-based weight-loss therapies that provide systemic metabolic unloading [125] and (ii) add-on antifibrotic strategies in patients with persistent fibrogenic activity despite reduced hepatic lipid stress [125,126].

#### 4.3.2. Semaglutide (GLP-1R Agonist): Systemic Metabolic Unloading with Histologic Efficacy

Semaglutide could be positioned as a whole-body metabolic backbone therapeutic rather than as a liver-restricted therapy. In the ESSENCE phase 3 interim analysis, semaglutide 2.4 mg resulted in statistically significant improvements in both primary histologic endpoints, including MASH resolution without worsening of fibrosis in 62.9% of patients, versus 34.3% with placebo, and fibrosis improvement without worsening of steatohepatitis in 36.8% of patients, versus 22.4% with placebo, at week 72. Mean body-weight change was −10.5% with semaglutide versus −2.0% with placebo, underscoring that its histologic benefit is tightly linked to broad systemic metabolic unloading [8]. Hence, the U.S. FDA has approved Wegovy (semaglutide) injection to treat MASH in adults with moderate-to-advanced fibrosis. However, patients with a history of thyroid cancer, with multiple endocrine neoplasia, T2DM, or with a known sensitivity to semaglutide are advised not to take this drug [127].

Mechanistic profiling further supports this interpretation, showing modulation of metabolic, inflammatory, and fibrosis-related pathways rather than a purely liver-restricted mode of action [113]. Accordingly, semaglutide functions as a systemic metabolic backbone therapy, particularly in patients with obesity, insulin resistance, or substantial cardiometabolic risk [8,111,113].

### 4.4. Near-Term Pipeline Organized by Mechanism Class

#### 4.4.1. Incretin and Multi-Agonist Strategies

Development beyond GLP-1 receptor mono-agonism largely reflects intensification of metabolic control rather than a fundamentally distinct therapeutic mechanism. In SYNERGY-NASH, the GLP-1R/gastric inhibitory polypeptide receptor (GIPR) dual agonist tirzepatide improved MASH resolution without worsening of fibrosis in 44–62% of treated patients versus 10% with placebo, with fibrosis improvement without worsening of MASH in 51–55% versus 30% [118] (Table 1). The GLP-1R/glucagon receptor dual agonist survodutide also improved MASH without worsening fibrosis and demonstrated reductions in liver fat content, along with favorable fibrosis-related biomarker changes in phase II studies [119]. These data support the use of dual agonists as potent metabolic backbone therapies capable of improving steatohepatitis activity and reducing hepatic lipid burden. However, patients with established fibrosis or persistent stromal activation may still require combination approaches incorporating direct antifibrotic therapies [111,118,119].

#### 4.4.2. Nuclear Receptor Modulators and Lipid Partitioning: PPAR and FXR Axes

Nuclear receptor pathways link metabolic regulation with inflammatory and stromal remodeling processes. The pan-PPAR agonist lanifibranor acts on metabolic, inflammatory, and fibrogenic pathways simultaneously and demonstrated histologic improvement in phase IIb studies [120]. However, lanifibranor may promote weight gain, suggesting that combination approaches with GLP-1RAs or other metabolic therapies may improve overall therapeutic benefit and tolerability [120]. These findings position pan-PPAR agonism as a potentially complementary strategy that combines metabolic modulation with direct effects on HSC activation and fibrosis progression [111,120,128].

#### 4.4.3. FGF21 Endocrine Mimetics: Stress-Resolving Metabolism with Emerging Histology Signals

Fibroblast growth factor (FGF) 21 is a stress-induced hormone produced in the liver and other metabolically active tissues [129]. FGF21 analogues are under development and represent a major non-incretin class currently adjacent to incretin-based therapies. In phase 2 trials, the two most advanced drug candidates pegozafermin and efruxifermin both reduced fibrosis without worsening of MASH and resolved MASH without worsening of fibrosis compared with controls (all *p* < 0.05) [107,121]. These agents act through systemic metabolic regulation and are being explored in patients with dyslipidemia or limited tolerance to incretin-based therapies [107,111,121]. For instance, Novo Nordisk just completed a 52-week phase IIb trial to assess the efficacy and safety of NNC0194 0499 as monotherapy or as combination therapy with semaglutide in patients with MASH and fibrosis stages F2 to F4 (NCT05016882) [130] and the SYMMETRY trial (Cohort D) showed tolerable profile of efruxifermin added to GLP-1RA appeared comparable to that of either drug alone, while also significantly reducing hepatic fat fraction and non-invasive markers of fibrosis in patients with MASH and diabetes type 2 [108]. Recently, Noureddin et al. published the data from an additional phase IIb trial investigating the efficacy and safety of efruxifermin in patients with compensated cirrhosis (stage 4 fibrosis). However, in patients with compensated cirrhosis caused by MASH, efruxifermin did not significantly reduce fibrosis at 36 weeks [122]. For now, no FGF21 analogs have been approved yet by the US FDA or the EMA. However, it remains a potential candidate for combination therapy with GLP1 agonists.

#### 4.4.4. Fatty Acid Synthase Inhibition and De Novo Lipogenesis

Fatty-acid synthase inhibition targets DNL upstream of lipotoxic inflammation. In a 52-week phase 2b trial, denifanstat produced a ≥2-point improvement in NAS without worsening of fibrosis in 38% of treated patients, compared with 16% with placebo, and MASH resolution with a ≥2-point NAS improvement without worsening of fibrosis in 26% versus 11% [123]. This mechanism reduces hepatocellular lipid synthesis through a pathway distinct from THR-β agonism or weight-loss pharmacotherapy [111,123].

#### 4.4.5. Precision Oligonucleotide Therapies: Genotype-Guided Hepatocyte Targeting

Genotype-directed therapies aim to target genetic drivers of disease heterogeneity. The *PNPLA3* I148M variant is the largest common genetic determinant of MASLD/MASH risk and progression [131]. Early clinical studies of *PNPLA3*-targeted RNA silencing and antisense approaches have demonstrated hepatic target engagement and reductions in liver fat in homozygous risk-allele carriers [132,133]. RNA interference targeting HSD17B13 has similarly shown substantial reductions in hepatic HSD17B13 mRNA, with encouraging early safety profiles [134]. Long-term therapeutic value will depend on confirmation of histologic benefit and integration with broader metabolic therapies [111,131,132,134].

#### 4.4.6. Direct Antifibrotic Strategies: Targeting Inflammation and Fibrosis

Direct antifibrotic drug development has been limited by pathway redundancy, stromal cell heterogeneity, and insufficient biomarkers of target engagement. HSCs contribute to both tissue repair and fibrosis, complicating strategies aimed at suppressing fibrogenic activity [76]. HSCs and Kupffer cells can be activated by release of DAMPs from dying hepatocytes and infiltrating pro-inflammatory macrophages. Therefore, reducing cell death in the liver seems to be an attractive liver-specific target, especially when patients reach their weight goals and get weaned off systemically acting weight control medication. Apoptosis signal-regulating kinase 1 (ASK1) is a ubiquitously expressed apical mitogen-activated kinase kinase kinase (MAP3K) that is activated by pathological stimuli in the human MASH liver, including oxidative and ER stress, and proinflammatory stimuli such as TNF-α and LPS [135,136]. Hence, the ASK1-p38/JNK pathway was found to be increased in livers of patients with MASH and preclinical evidence showed that ASK1 inhibition reduced liver cell death and fibrosis downstream of inflammatory signaling induced by NLRP3 [137,138]. However, translation into clinical application failed with the negative STELLAR trial, where selonsertib failed to improve fibrosis in patients with bridging fibrosis (F3, STELLAR3) or compensated cirrhosis (STELLAR [117]. Although selonsertib led to dose-dependent reductions in hepatic phospho-p38 expression indicating pharmacodynamic activity, it had no significant effect on liver biochemistry and progression to cirrhosis. As described earlier, infiltrating macrophages are a distinct feature of the inflamed liver. These cells can usually be distinguished from the resident Kupffer cells by their expression of CCR2, which is important for cell migration and attraction to inflammatory sites. However, these cells usually are more pro-inflammatory, and their metabolic capacity is reduced as opposed to Kupffer cells. Therefore, the novel, orally administered CCR2/CCR5 antagonist cenicriviroc was tested in patients with at-risk MASH (fibrosis II-III) and did not demonstrate antifibrotic efficacy in the AURORA (A Study for the Efficacy and Safety of CVC for the Treatment of Liver Fibrosis in Adults With NASH) phase III trial [116]. These results highlight the importance of patient selection, pathway relevance, and combination strategies in antifibrotic development [76,111,116,117]. It remains challenging to only address features of liver-specific pathologies without tackling systemic metabolic changes.

Because CCR2/CCR5 signaling and gut-derived PAMP exposure are closely linked to inflammatory cell recruitment, these strategies may be most relevant to portal and periportal immune trafficking, although prior phase III failure indicates that pathway targeting alone is insufficient.

#### 4.4.7. Gut Microbiome and Bile-Acid Axis

The gut microbiome contributes to metabolic and inflammatory signaling along the gut–liver axis. Altered NLRP3/NLRP6-IL-18 signaling can reshape the microbiota, increase portal delivery of TLR4 and TLR9 agonists, and amplify TNF-α-driven steatohepatitis progression [73]. Microbiome-directed therapies are therefore being explored as modulators of innate immune signaling and gut–liver inflammatory traffic [22,73].

#### 4.4.8. Zonation- and Niche-Targeted Antifibrosis

Recent work has expanded antifibrotic concepts beyond HSCs to include vascular and stromal niche interactions. A 2026 study identified ROCK2 upregulation in liver endothelial cells and perivascular stellate cells as a driver of vascular dysfunction and profibrotic signaling. The ROCK2 inhibitor TDI01 improved vascular phenotypes and reduced fibrosis in rodent and minipig MASH models, with early clinical studies reporting favorable pharmacokinetics and safety [124].

Experimental strategies targeting fibrogenic stromal cells are also being explored. In a 2026 study, anti-fibroblast activation protein alpha (FAP) chimeric antigen receptor (CAR) T cells generated in vivo via anti-CD5-targeted lipid nanoparticles transiently depleted profibrogenic HSCs and reduced fibrosis in murine MASH models [139]. Although still experimental, these approaches provide proof-of-principle that selective stromal targeting can modify fibrotic liver pathology [139,140].

Stromal and vascular-niche targeting is conceptually aligned with spatial fibrosis remodeling, but these approaches remain early and require validation against histological and clinical endpoints.

### 4.5. Combined Treatment Approaches

Combination therapy in MASH is increasingly explored to target complementary metabolic, inflammatory, and fibrogenic pathways. Earlier studies demonstrated that the combination of cilofexor (CILO), a non-steroidal farnesoid X receptor (FXR) agonist, and firsocostat (FIR), an acetyl-CoA carboxylase (ACC) inhibitor that reduces liver DNL, improved MASH activity and showed antifibrotic capacity in patients with advanced fibrosis and cirrhosis [141]. However, the FXR/ACC combination treatment has been associated with hyperlipidemia. Subsequently, a phase II trial aimed to mitigate these side effects by extending the drug combination. Here, semaglutide combined with firsocostat and/or cilofexor was well tolerated and produced significantly greater improvements in hepatic steatosis and non-invasive fibrosis markers compared with semaglutide alone [142]. Likewise, combining CILO/FIR with icosapent ethyl (Vascepa), an omega-3 fatty acid derivative, and fenofibrate was tested in patients with MASH with elevated triglycerides (≥150 and <500 mg/dL). In these patients, additional treatment with fenofibrate and icosapent ethyl was safe and effectively mitigated increases in triglycerides associated with acetyl-CoA carboxylase inhibition [143]. Network meta-analyses further suggest that the relative efficacy of combination regimens may vary by endpoint, with some therapies preferentially improving fibrosis regression while others more strongly promote MASH resolution [144].

Current treatment strategies generally begin with a metabolic therapy such as resmetirom, semaglutide, or a next-generation incretin and add mechanistically complementary agents targeting fibrosis, inflammation, or genetically defined susceptibility [7,8,141,144]. Because MASH is a systemic disease, treatment selection should also consider cardiovascular and renal outcomes in addition to liver histology [24,104].

### 4.6. Adult-Pediatric Gap in Zonation-Targeted MASH Therapy

Pediatric MASLD/MASH frequently shows periportal (zone 1) steatosis, inflammation, and fibrosis, whereas adult disease more often involves pericentral (zone 3) injury patterns [15,21]. However, mixed phenotypes are also seen, probably reflecting a shift towards a central-dominant phenotype during adolescence [145]. These age-dependent biological differences may influence the performance of adult-derived biomarkers (e.g., ALT or BMI), disease models, and lastly therapies in children. Hepatocyte-directed metabolic therapies optimized for pericentral lipotoxicity may not fully address periportal or ductular programs that are more prominent in pediatric disease [22]. In addition, liver zonation is not yet fully addressed by available medications. Current GLP-1 receptor agonists, such as semaglutide, have been studied in adults for the treatment of MASH and in children and adolescents over 12 years of age for obesity. However, they are currently approved by the FDA for the treatment of obesity, not MASH.

The clinical management of MASLD in children as well as in adults should take a holistic approach, addressing the full spectrum of mechanistic factors and comorbidities. The recommended primary treatment for adults and children alike is lifestyle interventions. For instance, restricting free sugar intake, for even just 8 weeks, decreased liver fat content as well as hepatic DNL [146]. However, although lifestyle modifications alone could improve or even resolve MASLD, a significant number of patients fail to achieve and maintain a meaningful bodyweight reduction.

Incretin-based pharmacotherapy is among the most mature metabolic interventions potentially applicable across age groups. Weghuber et al. published the results of applying a 2.4 mg dose of semaglutide once a week together with lifestyle interventions in adolescents (12–18 years). The combination treatment resulted in a greater reduction in BMI than lifestyle intervention alone [147]. Future pediatric research will require further age-specific endotype definitions and non-invasive biomarkers suitable for longitudinal monitoring. Here, ALT alone as an initial screening surrogate has been shown to be insufficient. A recent publication using metabolomics/lipidomics showed increased sensitivity to diagnose liver fibrosis in a cohort of Latino children with obesity, when dihydroxyacetone phosphate (DHAP), a marker of triglyceride synthesis, was combined with ALT [106]. However, this was a small early study, and DHAP needs to be validated in larger cohorts. Taking into account spatial differences between adults and children in MASH development, age might play an important role in evolutionarily conserved signaling pathways. For instance, Hedgehog signaling is increased and has been associated with portal-dominant injury, while centrilobular distribution of steatosis has been linked with Wnt/β–catenin signaling in a murine transgenic/knockout model [148]. The portal-dominant injury is accompanied by pronounced activation of immune cells residing close to the periportal fields. Hence, immune cell-directed therapies such as CCR2/CCR5 antagonism, likely in combination with systemically acting drugs, might have a benefit in some subgroups. However, this remains speculation without current evidence. Finally, genetics might also impact disease zonation patterns. In 822 US children (63% Hispanic) and among 60 candidate SNPs, rs738409C>G in *PNPLA3* was associated with both portal and lobular inflammation [149]. However, in 406 European children, this variant in *PNPLA3* was only associated with worse lobular inflammation [150]. More studies are needed in this area to verify these results, given the ongoing development of gene-targeted therapies.

### 4.7. Outlook: Toward Spatially Informed Precision Therapeutics

The next phase of MASH therapeutics will depend on the treatment architecture: identifying which patients to treat and when, defining reliable endpoints and investigating new surrogate endpoints for disease resolution, and selecting complementary mechanisms for combination therapy [5]. While the overarching acknowledgement of considering MASLD is not as an isolated liver disease, but the hepatic manifestation of a metabolic multisystem disorder, three priorities emerge from the current literature. First, trials should incorporate biomarker- and genotype-based stratification to improve patient selection and align therapies with the dominant drivers of disease. Second, treatment response assessment should expand beyond biopsy alone to include non-invasive biomarkers alongside histologic endpoints, as implemented in adult MASH trials. Third, therapeutic development will increasingly emphasize rational combination strategies rather than sequential monotherapy after treatment failure [116,141,142].

Precision oligonucleotide approaches targeting *PNPLA3* or *HSD17B13* illustrate how genotype-directed interventions may address specific drivers of disease heterogeneity [131,132,133,134]. Emerging stromal-directed strategies provide an additional experimental direction. In vivo anti-FAP CAR T therapy has shown that transient depletion of fibrogenic HSCs can reduce fibrosis and restore multicellular liver homeostasis in preclinical models. Although still experimental, these approaches illustrate a shift toward therapies matched to both molecular drivers and tissue context. Overall, the field is moving beyond single-agent proof-of-concept toward treatment strategies that match therapies to disease mechanisms and patient subtypes [131,132,134,139,151].

## 5. Conclusions

Metabolic liver disease is increasingly recognized as a spatially organized disease in which metabolic dysfunction, inflammation, and fibrogenesis evolve within distinct anatomical areas rather than throughout the liver. Recent advances in spatial transcriptomics, proteomics, lipidomics, imaging mass spectrometry, and multiplex imaging have substantially expanded the understanding of hepatic zonation, revealing how hepatocytes, LSECs, HSCs, macrophages, and other NPC populations coordinate disease progression across the porto-central axis. These technologies have shifted the field from describing isolated molecular pathways toward defining spatial disease programs, while also revealing that adult and pediatric disease follow distinct spatial trajectories that current tools and therapies do not yet fully address.

Current MASLD/MASH research, spatial and conventional alike, is constrained by biopsy sampling bias, technical variability across fixation and platform methods, transcript-protein discordance, and the still-unproven link between these findings and real clinical outcomes.

Ultimately, integrating hepatic zonation with multicellular signaling, metabolic remodeling, and tissue architecture provides a more comprehensive framework for understanding MASLD and MASH than conventional bulk analyses alone. As spatial technologies continue to mature and become increasingly integrated with clinical investigation, they are expected to facilitate the development of more precise biomarkers, improve patient stratification, and support the design of targeted therapeutic strategies tailored to the spatial and cellular heterogeneity of liver disease.

## Figures and Tables

**Table 1:** Adult-derived assumptions and pediatric applicability gaps in MASLD/MASH.

**Domain**	**Adult-Derived Assumption**	**Pediatric**	**Therapeutic/Trial Implication**
*Histology*	Zone 3 (pericentral) steatosis, hepatocyte ballooning, lobular inflammation, and perisinusoidal fibrosis [18].	Zone 1 (periportal) steatosis, portal inflammation, and portal fibrosis, with less ballooning [12,21].	Adult NAS-based endpoints may under-detect or mis-stage pediatric disease activity [12,18,21].
*Biomarkers/* *NITs*	ALT, BMI, MRI-PDFF, elastography, and serum fibrosis panels validated primarily in adult cohorts [24,104,105].	Pediatric thresholds and trajectories differ; ALT alone has insufficient predictive value for fibrosis [12,21,52,106].	Pediatric-specific biomarker validation and cutoffs are needed before adult NITs are applied unmodified [12,21,52,106].
*Drug* *Evidence*	Resmetirom and semaglutide efficacy data derive from adult F2-F3 MASH trials [7,8,107,108]	No drug is currently approved for pediatric MASH; GLP-1RAs are approved only for pediatric obesity [16,17].	Adult efficacy and dosing should not be directly extrapolated to children absent dedicated pediatric trials [16,17].
*Zonal* *Biology*	Therapeutic logic emphasizes pericentral lipotoxicity and perisinusoidal fibrogenesis [18,101].	Pediatric disease may be driven more by periportal, ductular, and immune-mediated programs [21].	Age- and spatially defined endotypes should inform pediatric trial design and target selection [16,17].
*Trial Design*	Adult trials rely on biopsy-confirmed F2-F3 disease with standardized histologic endpoints [109].	Pediatric biopsy cohorts are smaller and repeat biopsy is harder to justify longitudinally [110].	Longitudinal, non-invasive, pediatric-specific endpoints are needed to enable adequately powered trials [16,17].

Abbreviations: ALT, alanine aminotransferase; BMI, body mass index; GLP-1RA, glucagon-like peptide-1 receptor agonist; MASH, metabolic dysfunction-associated steatohepatitis; MRI-PDFF, magnetic resonance imaging proton density fat fraction; NAS, NAFLD Activity Score; NIT, non-invasive test.

**Table 2:** Approved and Phase III-tested MASH therapies, their primary biological targets, evidence tiers, histologic outcomes, and development-stage considerations. Regulatory approval sources are cited alongside pivotal trial data for resmetirom and semaglutide.

**Agent/** **Strategy**	**Primary Target/Pathway**	**Evidence Tier**	**Population Focus**	**Primary Histologic Endpoint(s) in Key Study**	**Met Primary Endpoint?**	**Key Limitation/Best Partner**
*Resmetirom* [7,114,115]	THR-β; hepatic lipid flux and mitochondrial oxidative capacity	Approved FDA (US); EC (EU)	Adults with biopsy-confirmed noncirrhotic MASH and F2-F3 fibrosis	MASH resolution without worsening of fibrosis; fibrosis improvement ≥ 1 stage without worsening of MASH	Yes	Limited direct antifibrotic activity; strong backbone for GLP-1/GIP combinations
*Semaglutide* [8]	GLP-1RA; weight loss, insulin resistance, adipose inflammation	Approved FDA (US)	Adults with biopsy-defined MASH and F2-F3 fibrosis	MASH resolution without worsening of fibrosis; fibrosis improvement ≥ 1 stage without worsening of MASH	Yes	GI tolerability; fibrosis response may remain incomplete in advanced disease
*Cenicriviroc* [116]	CCR2/CCR5; monocyte/macrophage recruitment	Phase III (negative)	Adults with MASH and F2-F3 fibrosis	Fibrosis improvement ≥ 1 stage without worsening of MASH	No	Development discontinued; highlights importance of patient enrichment and on-target immune readouts
*Selonsertib* [117]	ASK1; cellular stress signaling	Phase III (negative)	Adults with advanced fibrosis or cirrhosis due to MASH	Fibrosis improvement ≥ 1 stage without worsening of MASH	No	Lacked efficacy despite strong mechanistic rationale; underscores fibrosis pathway redundancy

Abbreviations: ASK1, apoptosis signal-regulating kinase 1; CCR2/CCR5, C-C chemokine receptor type 2/type 5; GLP-1RA, glucagon-like peptide-1 receptor agonist; MASH, metabolic dysfunction-associated steatohepatitis; THR-β, thyroid hormone receptor-β.

**Table 3:** Phase II and early/mechanistic MASH pipeline therapies, organized by mechanism class, with histologic outcomes and development-stage considerations.

**Agent/Strategy**	**Primary Target/Pathway**	**Evidence Tier**	**Population Focus**	**Primary Histologic Endpoint(s) in Key Study**	**Met Primary Endpoint?**	**Key Limitation/Best Partner**
*Tirzepatide* [118]	GIPR/GLP-1 dual receptor agonism	Phase II	Adults with biopsy-confirmed MASH and F2-F3 fibrosis	MASH resolution without worsening of fibrosis; fibrosis improvement ≥ 1 stage without worsening of MASH	Yes	Strong metabolic backbone candidate; may pair with THR-β agonists, FGF21 analogs, or antifibrotics
*Survodutide* [119]	GLP-1/glucagon dual receptor agonism	Phase II	Adults with biopsy-confirmed MASH and F1-F3 fibrosis	Improvement in MASH without worsening of fibrosis	Yes	Requires monitoring for lean-mass loss and tolerability; potential partner for hepatoselective lipid-modulating therapies
*Lanifibranor* [120]	Pan-PPAR agonism; lipid partitioning, inflammation, HSC modulation	Phase IIb	Adults with biopsy-confirmed, non-cirrhotic, highly active MASH; 76% had significant or advanced fibrosis, corresponding mainly F2-F3	SAF-A improvement ≥ 2 points without worsening of fibrosis; fibrosis improvement ≥ 1 stage	Yes	May cause weight gain; likely best combined with GLP-1-based metabolic therapies
*Pegozafermin/* *Efruxifermin* [107,121,122]	FGF21 analogues; systemic metabolic and hepatic lipid effect	Phase IIb	Adults with MASH and F2-F3 fibrosis; efruxifermin also evaluated in compensated MASH cirrhosis	Fibrosis improvement without worsening of MASH and/or MASH resolution without worsening of fibrosis	Yes	Durability and long-term fibrosis benefit under investigation; attractive partners for incretin-based therapies
*Denifanstat* [123]	FASN inhibition; suppression of DNL and hepatocellular lipid synthesis	Phase IIb	Adults with biopsy-confirmed MASH and F2-F3 fibrosis	≥2-point NAS improvement without worsening of fibrosis; MASH resolution with ≥2-point NAS improvement without worsening of fibrosis	Yes	Hepatocyte-directed strategy distinct from THR-β agonism or weight-loss pharmacotherapy; may complement systemic metabolic therapies
*TDI01* [124]	ROCK2; endothelial/perivascular vascular niche signaling	Early/Mechanistic (+Phase I)	Preclinical MASH models with early clinical translation	Preclinical fibrosis reduction and vascular normalization	Early clinical signals only	Requires controlled efficacy studies; may function best as add-on to metabolic backbone agents

Abbreviations: FASN, fatty acid synthase; FGF21, fibroblast growth factor 21; GIPR, gastric inhibitory polypeptide receptor; GLP-1RA, glucagon-like peptide-1 receptor agonist; HSC, hepatic stellate cell; MASH, metabolic dysfunction-associated steatohepatitis; NAS, NAFLD Activity Score; PPAR, peroxisome proliferator-activated receptor; ROCK2, Rho-associated coiled-coil containing protein kinase 2; SAF-A, steatosis–activity–fibrosis activity score; THR-β, thyroid hormone receptor-β.

## Data Availability

No new data were created or analyzed in this study. Data sharing is not applicable to this article.

## Abbreviations

ACC	acetyl-CoA carboxylase
ACTA2	actin alpha 2
ADH	alcohol dehydrogenase
AHR	aryl hydrocarbon receptor
ALB	albumin
ALDOB	aldolase B
ALT	alanine aminotransferase
AMPK	AMP-activated protein kinase
APOA1	apolipoprotein A1
APOC2	apoliprotein C2
ARG1	arginase 1
ASK1	apoptosis signal-regulating kinase 1
ASS1	argininosuccinate synthase 1
ATP	adenosine triphosphate
BMP	bone morphogenetic protein
BMI	body mass index
CAR	chimeric antigen receptor
CCL	C-C motif chemokine ligand
CCR2/CCR5	C-C chemokine receptor type 2/type 5
ChREBP	carbohydrate-responsive element-binding protein
CYP	cytochrome P450
CYP1A2	CYP family 1 subfamily A member 2
CYP3A4	CYP family 3 subfamily A member 4
DAMPs	damage-associated molecular patterns
DCA	deoxycholic acid
DNL	de novo lipogenesis
ECM	extracellular matrix
ER	endoplasmic reticulum
EV	extracellular vesicle
FABP4	fatty acid-binding protein 4
FAP	fibroblast activation protein alpha
FASN	fatty acid synthase
FFA	free fatty acid
FGF21	fibroblast growth factor 21
FoxO1	forkhead box protein O1
FXR	farnesoid X receptor
G6PC1	glucose-6-phosphatase catalytic subunit 1
GIPR	gastric inhibitory polypeptide receptor
GLP-1RA	glucagon-like peptide-1 receptor agonist
GLS2	glutaminase 2
HAL	histidine ammonia-lyase
HCC	hepatocellular carcinoma
HNF4α	hepatocyte nuclear factor 4 alpha
HSC	hepatic stellate cell
HSD17B13	hydroxysteroid 17-beta dehydrogenase 13
IAA	indole-3-acetic acid
ICAM1	intercellular adhesion molecule 1
IL	interleukin
IPA	indole-3-propionic acid
JNK	c-Jun N-terminal kinase
LAM	lipid-associated macrophage
LGR4/5	leucine-rich repeat-containing G-protein coupled receptor 4/5
LIPC	hepatic lipase
LPS	lipopolysaccharide
LSEC	liver sinusoidal endothelial cell
MAPK	mitogen-activated protein kinase
MASLD	metabolic dysfunction-associated steatotic liver disease
MASH	metabolic dysfunction-associated steatohepatitis
MBOAT7	membrane-bound O-acyltransferase domain-containing 7
MHC-II	major histocompatibility complex class II
MRI-PDFF	magnetic resonance imaging proton density fat fraction
MS	mass spectrometry
MT1A	metallothionein 1A
MUFA	monounsaturated fatty acid
NAFLD	non-alcoholic fatty liver disease
NASH	non-alcoholic steatohepatitis
NAS	NAFLD activity score
NF-κB	nuclear factor-kappa B
NKD1	naked cuticle homolog 1
NLRP3	nucleotide-binding oligomerization domain-like receptor family pyrin domain-containing 3
NPC	non-parenchymal cell
NOTUM	palmitoleoyl-protein carboxylesterase NOTUM
OAT	ornithine aminotransferase
OXPHOS	oxidative phosphorylation
PAMPs	pathogen-associated molecular patterns
PCK1	phosphoenolpyruvate carboxykinase 1
PNPLA3	patatin-like phospholipase domain-containing protein 3
PPAR	peroxisome proliferator-activated receptor
ROS	reactive oxygen species
RSPO3	R-spondin 3
SAF	Steatosis–activity–fibrosis
SCFA	short-chain fatty acid
SDS	serine dehydratase
SFA	saturated fatty acid
SOCS1	suppressor of cytokine signaling 1
SREBP-1c	sterol regulatory element-binding protein 1c
T2DM	type 2 diabetes mellitus
TCA	tricarboxylic acid
TGF-β	transforming growth factor beta
THR-β	thyroid hormone receptor beta
TLR	Toll-like receptor
TMAO	trimethylamine N-oxide
TM6SF2	transmembrane 6 superfamily member 2
TNF-α	tumor necrosis factor alpha
VAT	visceral adipose tissue
VLDL	very-low-density lipoprotein
WNT	wingless-related integration site
YAP/TAZ	yes-associated protein/transcriptional coactivator with PDZ-binding motif
ZO-1	zonula occludens 1
ZNRF3	zinc and ring finger protein 3
